# Using a Community-Engaged Research (CEnR) approach to develop and pilot a photo grid method to gain insights into early child health and development in a socio-economic disadvantaged community

**DOI:** 10.1186/s40900-017-0078-7

**Published:** 2017-12-18

**Authors:** Emma Lowrie, Rachel Tyrrell-Smith

**Affiliations:** Centre for Early Child Development, Blackpool Better Start (NSPCC), 1 Bickerstaffe Square, Blackpool, FY1 3AH UK

**Keywords:** Community development, Community engaged research, Co-production, Public involvement, Early child development

## Abstract

**Plain English summary:**

This paper reports on the use of a Community-Engaged Research (CEnR) approach to develop a new research tool to involve members of the community in thinking about priorities for early child health and development in a deprived area of the UK. The CEnR approach involves researchers, professionals and members of the public working together during all stages of research and development.

Researchers used a phased approach to the development of a Photo Grid tool including reviewing tools which could be used for community engagement, and testing the new tool based on feedback from workshops with local early years professionals and parents of young children.

The Photo Grid tool is a flat square grid on which photo cards can be placed. Participants were asked to pace at the top of the grid the photos they considered most important for early child health and development, working down to the less important ones at the bottom. The findings showed that the resulting Photo Grid tool was a useful and successful method of engaging with the local community. The evidence for this is the high numbers of participants who completed a pilot study and who provided feedback on the method. By involving community members throughout the research process, it was possible to develop a method that would be acceptable to the local population, thus decreasing the likelihood of a lack of engagement. The success of the tool is therefore particularly encouraging as it engages “seldom heard voices,” such as those with low literacy.

**Abstract:**

**Background:**

The aim of this research was to consult with professionals and parents to develop a new research toolkit (Photo Grid), to understand community assets and priorities in relation to early child health and development in Blackpool, a socio-economic disadvantaged community. A Community–Engaged Research (CEnR) approach was used to consult with community members. This paper describes the process of using a CEnR approach in developing a Photo Grid toolkit.

**Methods:**

A phased CEnR approach was used to design, test and pilot a Photo Grid tool. Members of the Blackpool community; parents with children aged 0–4 years, health professionals, members of the early year’s workforce, and community development workers were involved in the development of the research tool at various stages. They were recruited opportunistically via a venue-based time-space sampling method. In total, 213 parents and 18 professionals engaged in the research process.

**Results:**

Using a CEnR approach allowed effective engagement with the local community and professionals, evidence by high levels of engagement throughout the development process. This approach improved the acceptability and usability of the resulting Photo Grid toolkit. Community members found the method accessible, engaging, useful, and thought provoking.

**Conclusions:**

The Photo Grid toolkit was seen by community members as accessible, engaging, useful and thought provoking in an area of high social deprivation, complex problems, and low literacy. The Photo Grid is an adaptable tool which can be used in other areas of socio-economic disadvantage to engage with the community to understand a wide variety of complex topics.

**Electronic supplementary material:**

The online version of this article (10.1186/s40900-017-0078-7) contains supplementary material, which is available to authorized users.

## Background

What a child experiences during the early years usually provides a trajectory for the rest of their life [1, 2]. In particular, a young child’s development is profoundly affected by their early care-giving experiences. In neighbourhoods where parents face multi-level complex problems such as substance misuse, mental ill health or intimate partner violence, children are affected too. Exposure to high levels of early adversity and toxic stress through increased allostatic load predisposes children to problems in learning, behaviour and health across their life course [3–7].

Blackpool is currently the most deprived of all 326 local authorities in the UK [8]. Across the town there are high levels of domestic violence, alcohol related hospital admissions and mental ill-health which is further compounded by low educational attainment and literacy levels. Blackpool has the highest rate of looked after children in the UK (164 per 10,000) as well as high levels of child abuse and neglect [9]. Children growing up in Blackpool have some of the worst outcomes in the UK. In April 2015 Blackpool Better Start was allocated £45 million over 10 years from the Big Lottery Fund with the aim to improve outcomes for children from conception to 3 years in three key areas: language and communication, social and emotional development, and diet and nutrition. The initiative aims to use early intervention focused on prevention to improve the health and developmental outcomes of young children at two developmental milestones; healthy gestation and birth, and school readiness.

In order to support families and children living in communities like Blackpool, high quality, effective, evidence-based programmes should be implemented. However, implementing a suite of programmes and increasing access to services and resources is often not enough to substantially change child health and developmental outcomes [10]. The most successful initiatives tend to have the following characteristics: they address multiple social determinants of health, utilise community development approaches to tailor and align interventions to community assets and priorities [11–14]; have shared goals between partners [15] and use collaborative methods to build trust and generate appropriate change [16, 17]. In order to select, develop and implement a suite of interventions to address early child health and development in Blackpool, it was important to first understand community needs, priorities and readiness for change. By utilising community engagement, a culture conducive to long-lasting change and an effective shift towards improved child outcomes can be created.

In the UK, many public health interventions which aim to improve health or reduce health inequalities are now involving the community in programme design and development [18–20]. However, in areas of high need, researchers often find it difficult to engage and collaborate with the community, particularly when using traditional methods unsuitable for low literacy populations [21, 22]. A review of several research methods deemed them unsuitable for engaging with the Blackpool community. The current paper describes the process of using a Community-Engaged Research (CEnR) approach to develop an acceptable, visual and pragmatic tool (Photo Grid) to understand local needs, priorities and readiness for change.

## Methods

### Design

Community-Engaged Research (CEnR) principles were used to develop and test a Photo Grid as a research and engagement tool. CEnR has become increasingly popular across philanthropic organisations, academic institutions and governmental domains [23] as it requires partnership development, co-operation, and a commitment to addressing local community issues [24–26]. An overview of CEnR principles is presented in Table 1 (adapted from [27–29]).

**Table 1:** Community-Engaged Research principles

Research Stage	Community-Engaged Research Principles	How Principle Used
Research Objective	Community input in identifying locally relevant issues and understanding priorities and assets	Aim of research to develop a toolkit to allow for identification of local priorities
Study Design	Researchers work with the community to ensure the study design is feasible and culturally acceptable in terms of content	Development of study design – Phase 2
Recruitment & Retention	Researchers consult with community representatives on recruitment and retention strategies	Local professionals and parents consulted on recruitment strategies in Phase 2
Instrument Design	Instruments adopted from other studies are tested/adapted to fit local populations and their needs	Community involvement involved in method development in Phases 1 and 2
Data Collection	Community members involved in some aspects of data collection, gaining skills and knowledge of research methods	Some parents (*n* = 3) involved in Phase 2 development were trained and involved in Phase 3 data collection
Analysis & Interpretation	Researchers share the results of the analysis with community members for comments and interpretation	Yes, not reported in this paper
Dissemination	Results disseminated in community venues as well as in peer reviewed journals and presented to frontline workers with community support	Yes, not reported in this paper
Further Commitment	A continued partnership between researchers and the community to use findings to advocate for change, enhance local resources and improve local practice	Continued community engagement and promising sense of partnership and shared understanding

Using a CEnR research design resulted in a three phase development plan. In Phase 1, the research team reviewed existing literature and presented identified research methods to local professionals. Further information will be provided on participants in the following section. The most beneficial aspects of each method and the barriers each might present to community participation were identified by the group. An initial list of factors considered to be most important for early child health and development locally was drawn up by local professionals allowing for the initial development of the Photo Grid and accompanying materials, in line with local population needs. Local community members were not included in Phase 1 to ensure any factors that could cause distress were removed or reframed appropriately. In Phase 2, local community members and members of the early year’s workforce were asked to trial a prototype Photo Grid and provide feedback based on their own experiences and local knowledge. The toolkit was then adapted accordingly. Participants in this Phase also provided advice and support with recruitment and data collection for Phase 3. In Phase 3, the Photo Grid was piloted within local venues with community members. Feedback was gained with regards to the engagement, understanding and value of the Photo Grid.

### Participants

Phase 1 participants were a convenience sample of health professionals (midwives), psychologists and community development workers who were approached in the workplace (*n* = 10). In Phase 2, five participants were recruited from a local parent group. Each parent expressed an interest in early child health and development, had a child aged 0-4 years had lived in Blackpool for a minimum of 5 years. They were asked to participate in a demonstration, discussion and development group to look at the new way of engaging local families in the Better Start initiative. Also participating in Phase 2 were eight members of the early years workforce, recruited from local children’s centres. They were asked to participate in the development of a new community engagement tool which would be piloted in their setting. Each had worked extensively in the Blackpool community (minimum 3 years) and could provide widespread knowledge about local families with young children. In Phase 3, venue-based time-space sampling [30] was used. This is a probability-based strategy for recruiting members of a target population congregating at specific locations and times. In total208 individuals from children’s centres and other early years settings (e.g. faith-based toddler groups) were asked to take part in an activity looking at priorities for early child health and development. Substantial interest in the activity allowed the target sample of 200 participants to be exceeded. Most participants (*n* = 188) provided feedback on the Photo Grid tool, a response rate of 90.4%. A small incentive of refreshments was offered to participating community members as a token of gratitude for their time, energy and resources [31]. Opportunities to pilot the tool and provide insights into priorities for early child health and development were promoted using posters/leaflets distributed within the children’s centres, an advert placed in the local community newspaper and via social media [32, 33]. Table 2 presents the demographic characteristics of the Phase 3 participants. The majority of participants were female (91%) and currently had a child aged 3 years or under (70%) thereby falling within the Blackpool Better Start population of interest. However, young parents and fathers were under represented in the sample with only 1% of participants aged 20 years or under and 9% males.

**Table 2:** Participant demographic information (Phase 3)

		Frequency (n)	Percentage (%)
Gender	Female	175	91.2
	Male	17	8.9
Single Parent	No	134	72.4
	Yes	51	27.6
Age	Under 20 years	2	1.1
	20–24 years	31	16.3
	25–29 years	54	28.4
	30–34 years	41	21.6
	Over 35 years	62	32.6
Employment Status	Employed	93	48.4
	Unemployed	99	51.6
Number of Children	None	3	1.6
	One	52	27.8
	Two	65	34.8
	Three	36	19.3
	Four	13	7.0
	Five or more	18	9.6
Age of Youngest Child	Pregnant	12	6.5
	Under 6 months	16	8.7
	6–12 months	26	14.1
	12–24 months	37	20.0
	2 years	24	13.0
	3 years	15	8.1
	4 years	14	7.6
	5 years or over	41	22.2

## Results

### Phase 1: Photo grid development with professional workers

To explore the community needs, priorities and readiness for change with regards to early child health and development three “traditional” research methods were discussed in an meeting by a group of professional workers. These were: Q-Methodology [34, 35]; Rank Order Methods [36]; and Photo-Elicitation [37]. Q-Methodology uses a sorting technique to examine “points of view” around a topic. Participants are grouped by similar opinions. Rank Order Methods involve participants placing a set of items in some form of order. The measure of order can include liking, effectiveness, importance etc. Photo-elicitation is a method of interviewing which uses visual images to elicit information from participants. These were selected owing to their pragmatic and simple nature, ability to be used within a variety of settings and populations, whilst providing rich, detailed information without being burdensome [38, 39]. The benefits and challenges presented by each method were considered with the population of Blackpool in mind. Findings from these discussions are summarised in Table 3.

**Table 3:** Professional feedback on three traditional research methods

Method	Positive	Negative
Q-Methodology	Will elicit a good understanding of community views of factors associated with early child health and development.	Too many statements will be challenging in a low literacy population. It will require a lot of work in a short time frame.
	Good grid structure, simple and clear to understand.	Need additional software for analysis (Uses R-Methodology).
	“Think-out-loud” protocol is desirable. It allows for the reasoning behind factor placement to be captured.	Data analysis groups participants according to similar viewpoints not exploratory in nature.
Rank Order Methods	Less complex data analysis than Q-methodology. Useful for initial instrument development.	No grid. It does not allow for any topics to be given an equal weighting.
	Easy to understand. There is a simple linear structure associated with priority or preference ranking.	Not as interesting or engaging as the other two research methods for participants. We want them to want to take part in future projects.
Photo-Elicitation	Less text, images allow for individuals own interpretation of the aim. As the study is exploratory this may be helpful.	Less stringent research method. Will data gained still be worthwhile with a lack of numerical data.
	Images generally make difficult topics more accessible and easier to discuss. Broaching them is less probing than asking direct questions.	Timeline too short to allow participants to take their own photos (i.e. Photo-Voice). Images would need to be pre-generated.

The decision was made to develop a new research and engagement tool in order to best fit the local population and their needs. The resulting Photo Grid amalgamated the most beneficial features of each “traditional” method. The simple structure of the grid and cards were utilised from Q-methodology. Photo cards were proposed to represent factors associated with early child health and development. Participants in this phase strongly advocated for the use of images rather than statements on the cards to account for the low literacy levels of the target population. It was thought that the interactive process of placing the cards on a large grid would be an engaging method. The simple linear data coding of the rank order method was applied to the grid to gain and understanding of common needs and priorities across the community (method and results not reported here), a significant barrier to participation for both service access and research previously. Lastly, a “think-out-loud” protocol was adopted to capture each individual interpretation of images and positioning of cards on the Photo Grid, a method anticipated to increase comfortable disclosure allowing a relationship to be built between facilitators and community members.

Five key areas of early child health and development: healthy gestation and birth, social and emotional development, language and communication, diet and nutrition, and school readiness; were used to generate a long list of factors (*n* = 60). The list was streamlined following discussions with Phase 1 participants where factors representing similar factors were combined and factors with the potential to be emotive were removed (e.g. parental drug use and intimate partner violence). Following this iterative process, 37 factors remained.

Photo cards were designed to represent each of these 37 factors; the front of each card had a title and an image representing the factor, the reverse included a standardised definition intended for use if participants required further explanation or examples. Following further discussions, three factors were combined/ removed and a previously unconsidered factor was added to the set. Three images were changed in order to ensure consistency with current health messaging and advice. The titles of the final 35 cards can be seen in Additional file 1.

To complete the toolkit, a minimal set of non-identifiable demographic questions (gender, age, single parent status, no. of children and age of youngest child) were included as tick boxes at the side of the Photo Grid. The tool was designed using PVC coated cardboard, to make each grid reusable. Photographs of each completed Photo Grid were taken as a record of the card placement and demographic information before being wiped clean. A short instruction sheet and verbal guidance was designed to standardise the information provided to participants. A recording sheet was designed to enable facilitators to record conversation details eliciting valuable qualitative insights. This included four open-ended questions which enquired about (1) the ordering of the cards, (2) the relevance of the factors, (3) opinions about the usefulness of the Photo Grid tool as a research and engagement tool and (4) provided the opportunity for any other information to be provided. These were linked to the corresponding photographs using a unique identifying number.

### Phase 2: Photo grid testing and adaption with (a) local parents group members and (b) early years workers

Five local parents participated in a demonstration, discussion and development group to test and feedback on the resulting prototype Photo Grid. Three main pieces of positive feedback emerged: (1) the activity was interesting, enjoyable, and prompted group discussion regarding early child health and development priorities; (2) the use of images on cards made it easy to discuss the factors in an unassuming manner; and (3) there was appreciation for the proposed wipe clean, re-useable design of the Photo Grid.

**Fig. 1:**
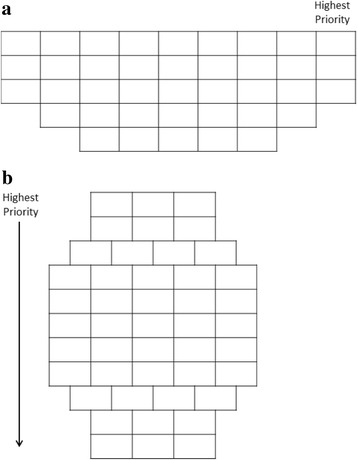
**a** Traditional Q-Grid. **b** Modified Photo Q-Grid

Two areas for improvement were identified: (1) Initially, the number of cards was overwhelming. Working through this issue, the task was made more manageable by adding a sorting step to the protocol. This involved sorting the cards into high, middle and low priority groups prior to placing the cards on the grid. (2) The prototype adopted a traditional Q-Grid layout where cards are placed from left to right, low to high priority (Fig. 1a). Participants found this layout confusing, opting to place cards from top to bottom with cards reflecting a higher priority placed at the top of the Photo Grid. In order to ensure ease and consistency in completion of the task, the orientation of the Photo Grid was changed and a directional arrow added for clarity (Fig. 1b). The instruction sheet and verbal guidance were modified to complement the protocol changes.

Following these modifications, the process was repeated with eight early years’ workers. They confirmed the suitability of the activity for use with the local community, reiterating similar positive feedback to that cited by the local parents group members. Additionally they considered the inclusion of each card based on the appropriateness of the image, title and explanatory statement. At this point, three cards were edited for clarity of wording and a further demographic question (employment status) was added to the side of the Photo Grid tool.

Three of five local parents involved in Phase 2 were interested in continued involvement in Phase 3 of the research. Following the CEnR approach, they were trained by the research team in data collection protocols and were volunteer facilitators in Phase 3. This allowed parents to gain first-hand experience of research, broaden their skill sets and increased the capacity for study recruitment and data collection.

### Phase 3: Photo grid pilot within local community members in children’s settings

The aim of Phase 3 was to pilot the Photo Grid toolkit with the local community. Participants were asked to individually complete the Photo Grid so that it represented the most important factors for early child health and development for themselves and the local community, and provide feedback on the Photo Grid as a research and engagement tool. The majority of participants (73.5%) agreed the Photo Grid was a good engagement tool and would elicit an overview of community priorities for early child health and development. Some participants commented that they enjoyed completing the activity (11.5%), with three specifically attributing this to the use of images rather than words. Many participants (35.5%) described how the task had allowed them to “think again” about priorities they had for their own children and use the time to reflect on what they believed to be important. Conversely, there were a small number of participants (2%) who did not find the activity useful stating that they were already confident in their own priorities as a parent.

Although participants were encouraged to complete the grid independently, a small number completed the task in pairs/groups (4.5%). On these occasions, the cards provided prompted discussion as groups debated each factor before coming to consensus on high, middle and low priorities. Participants were happy that the Photo Grid allowed the opportunity for their opinions about what action is needed locally to be heard and recognised that it gave them chance to learn more about early child health and development.

Approximately a quarter of participants provided further information about the ease of completing the task and/or supplied suggestions of improvements for future research. These participants felt that many of the cards were top priorities and would have liked to the option to place a greater number of cards at the top of the grid. They highlighted a need for greater specificity around the age of the child as the pregnancy period was often prioritised over early infancy. Participants noted that their priorities differed depending on their child’s age and development stage, behaviour, and personality characteristics. This feedback has since been incorporated into the toolkit instructions. A small number of participants (3.5%) found the task challenging, the number of cards overwhelming and the more conceptual cards difficult to comprehend. Additional facilitator support was provided to these individuals who subsequently reported enjoyment upon completing the Photo Grid.

## Discussion

The current paper describes the process of using a Community-Engaged Research (CEnR) approach to develop an acceptable, visual and pragmatic tool (Photo Grid) to understand the Blackpool community needs, and priorities for early child development. A CEnR approach involves researchers, professionals and members of the public working together during all stages of research and development.The tool was seen by community members as accessible, engaging, useful and thought provoking in an area of high social deprivation, complex problems, and low literacy.

Using a CEnR approach proved to be effective evidenced by the numbers engaged in the development and pilot phases, some of whom remained engaged throughout taking on volunteer facilitator roles. By involving professional workers, parents, early years workers and community members in the development and testing of the Photo Grid the usability and appropriateness of the instrument was maximised. Participants were able to engage with researchers in a meaningful way, providing valuable insights into local needs and priorities around early child health and development. The CEnR approach allowed for a mutually beneficial partnership to form between research staff and local community members. By involving parents and the community at each stage of the research (i.e. tool kit development, trained parent facilitators, and dissemination), there are promising signs that a culture of trust and collaboration is in its early stages of development.

In order to mitigate any potential distress a decision was taken to not involve community members in Phase 1 of the research. There was concern that the discussion of including potentially emotive factors (e.g. intimate partner violence, parental substance misuse) may cause upset to those who have experienced them directly. Upon reflection, it may have been beneficial to involve community members in this stage of the research. As part of a collaborative approach representatives from the community should be considered equally able to decide what may, or may not cause feelings of distress. Future research using the Photo Grid method to investigate other areas of interest should consider involving community members as early as possible. However, all participants should be made aware that potentially distressing topics may be discussed and provided with a list of support services as a precaution.

As with many community-based projects, recruitment and sampling were a limiting factor. In particular, fathers and young parents were under-represented in all phases of the Photo Grid development and testing. A venue-based time-sampling method gave opportunistic yet resourceful access to community members. However, those who do not engage with services were subsequently not involved in the tool design and piloting. Future research should examine if the Photo Grid tool is as successful in engaging the unengaged.

In addition, whilst using a CEnR framework was effective, community involvement in research can be measured on a continuum. Community-Based Participatory Research (CBPR) forms the ideal or gold standard of the approach aiming for a full partnership between researchers and the community in all areas of research design including shared ownership of materials developed and joint interpretation of findings [23]. Whilst powerful and conducive to creating a culture of understanding and positive change, this is difficult to achieve and requires the development and maintenance of long-term relationships [26, 40, 41]. Until these relationships are built, utilising a CEnR framework is considered most effective.

As this method of interacting with the community has been successful, it will be used to gain a more in depth understanding of individual topics in more detail throughout the span of Blackpool Better Start. Forthcoming work will utilise the findings from the Photo Grid analysis (not reported) to tailor the development and implementation of programmes to suit the local context. The Photo Grid tool appears reliable with feedback about the tool relatively consistent across all participants. Future research utilising the tool in another area with similar deprivation and literacy levels may enhance its reliability. Similarly, the Photo Grid tool was successful in engaging participants in research and eliciting discussions around important factors associated with early child health and development. This suggests that the tool is a new, successful method of gaining information on this subject. Future research adapting the tool to prompt community discussion around other topics will allow for a further assessment of its validity. It is hoped that other researchers can learn from the CEnR process detailed in this paper and utilise the Photo Grid method. It has potential for adaptation and could be used as an effective tool to examine a wide range of topics in other areas of high socio-economic disadvantage and low literacy levels.

## Conclusions

In conclusion, the Photo Grid toolkit was seen by community members as accessible, engaging, useful and thought provoking in an area of high social deprivation, complex problems, and low literacy. Involvement of the community in the development of the tool was seen as an enabler to this success, particularly with a population considered to contain many “seldom heard voices”. The Photo Grid is an adaptable tool which can be used in other areas of socio-economic disadvantage to engage with the community to understand a wide variety of complex topics.

## Additional file

Additional file 1: It is acknowledged that the final set of cards is not a complete representation of all factors conducive to early child health and development, but rather a starting point for conversation with the community allowing us to better understand community priorities. (DOCX 11 kb)

## Abbreviations

CBPR: Community-Based Participatory Research
CEnR: Community-Engaged Research
