# Supplementary material for: Using a Community-Engaged Research (CEnR) approach to develop and pilot a photo grid method to gain insights into early child health and development in a socio-economic disadvantaged community
Source: Res Involv Engagem. 2017 Dec 18;3:29. doi: 10.1186/s40900-017-0078-7 (PMC5733929; doi:10.1186/s40900-017-0078-7)
Supplement: Additional file 1: — It is acknowledged that the final set of cards is not a complete representation of all factors conducive to early child health and development, but rather a starting point for conversation with the community allowing us to better understand community priorities. (DOCX 11 kb) [file 40900_2017_78_MOESM1_ESM.docx]

**List of factors associated with early child health and development for use on Photo Grid cards**

| **Healthy Gestation & Birth** | **Social & Emotional** | **Language & Communication** | **Diet & Nutrition** | **School Readiness** |
| --- | --- | --- | --- | --- |
| No Alcohol in Pregnancy | Protection | Playing Together | Smoke Free Home | Early Learning Milestones |
| No Smoking in Pregnancy | Feeling Safe & Secure | Sharing Books, Stories and Rhymes | Good Diet for Child | Good Behaviour |
| Good Mental Health in Pregnancy | Parent Child Bonding | Playing with other Children | Tooth Brushing | Fine Motor Skills |
| Healthy Diet in Pregnancy | Support from Family or Caregivers | Age Appropriate Interaction | Less Junk Food for Child | Physical Coordination |
| Professional Services in Pregnancy | Building Better Brains | Serve & Return Interaction | Only Milk or Water to Drink | Able to Follow Instructions |
| No Stress in Pregnancy | Dad’s or Male Role Models | Starting to Talk | Outdoor Space | Pre-School Childcare |
| Breastfeeding | Appropriate Discipline | Technology Free Time | Less Time Sat Down | Toilet Trained |
